# English Impudence and American Servility

**Published:** 1857-05

**Authors:** 


### EDITORIAL DEPARTMENT.

---

*English Impudence and American Servility.*—Any hesitation which we may have felt in the selection of a subject for an editorial article for this number, is thoroughly removed by the kindness of a friend, who has pointed out to us the following passage from an essay on the "Effects of Climate on Tuberculous Disease;" By Edwin Lee, M. R. C. S., London, being the Dissertation to which the Fiske Prize Fund was awarded, June 6, 1855. Published by request of the Rhode Island State Medical Society. The passage appended seems to us particularly worthy of comment, in whatever point of view we may regard it:

"A minute examination of physical signs supplied by an abnormal state of the respiratory function, would, in many instances, lead to the discovery of tubercles in the lungs before their presence was indicated by clearly marked symptoms; but the majority of practitioners are scarcely capable of making such an examination as would frequently suffice to detect pulmonary disease in its earliest stage, especially in England, *and probably also in America, where exploration of the state of the organs contained in the thoracic cavity, by means of auscultation and percussion, forms no part of the education of medical students.*"

(Italics our own.)

The circumstances under which this remarkable assertion comes to us are these: The Rhode Island Medical Society has charge of a prize fund named after its honored founder, the Fiske Prize Fund. It offered a prize of one hundred dollars for the best essay on the effects of climate on tuberculous disease, and awards the prize in usual form to Mr. Edwin Lee, F. R. C. S., London. Mr. Lee of course wrote for this prize, with the expectation that a committee of Americans would adjudge upon the merits of his composition. Now if it were true that physical exploration is unknown among the medical profession of this country, and "forms no part of the education of medical students" here, it strikes us that it was a very injudicious and impudent thing in Mr. Lee to say so. Assuming that his premises were correct, it was the duty of this gentleman, in the prosecution of his subject, to inform us so far as in him lay, to enlighten our ignorance by describing to us the shape of the old fashioned stethoscope—he himself had never heard of Cammann's instrument—and give us a few brief instructions on how to use it. Then he should have gone on to explain percussion to us, illustrating it by the familiar half-empty beer-barrel which has so long served as the comparison for this purpose.

We should then have been able to have recognized in the essayist a man whose mission it is to teach; who, himself erudite, showers down the blessing of his scholarship on benighted America. As it is, he simply says, "you do not know enough to appreciate me, and I can only inform you of your ignorance."

So far we have given Mr. Lee the most comfortable horn of the dilemma. Now to examine the truth of his assertions. He says, in the first place, that the generality of practitioners in England are incompetent to recognize the early signs of tubercle. We presume he knows, at anyrate we are not inclined to dispute him, and think it altogether probable.

But again he tells us that the same is true of American practitioners—that they, too, are incompetent to physical exploration. We can form no opinion as to whence Mr. Lee obtained his information on this point. We are not aware that he has ever traveled in America, and met the "generality of American practitioners" at the bedside to ascertain the facts. But we do know that the generality of American practitioners have a fair knowledge of physical exploration, and we could point out to him very many country practitioners whose diagnosis is accurate and reliable in diseases of the chest. This statement may or may not cover the majority of physicians in town and country—there are differences here as in every thing else—but it should be borne in mind that physical exploration is a new science; and, for ourselves, it is to us a matter of wonder that it should so rapidly have become so widely known among the profession. Moreover, if Mr. Lee has studied the literature of the subject, he should be aware that America has produced some of the earliest and best works on physical exploration. There is such a singular combination of impudence and ignorance in his bold assertion, that we hardly know whether to be amused or indignant. On mature reflection, we have concluded to laugh at him, and reserve our indignation for those meekest of the meek, the committee which awarded this prize.

But before turning to them we wish to refer to another aspect of this assertion. It has been the fashion with writers to say much about the difficulties of physical explorations; and its intricacy has been magnified until many are deterred from its prosecution, or led to an undue want of reliance on their own judgment. We know of but one exception to this remark, and that is Prof. Flint; who, in his admirable work has the honesty and courage to say that the practice of the art is not so difficult as to defeat the diligence of any student with a tolerable ear, who will read the standard authors on the subject and practice as much as possible under their direction.

But so long as we throw a vail of mystery around it, so long as we hold out the idea that only a favored few are competent to its practice, we shall retard the progress of science and the diffusion of its blessings. We do not mean to imply that a *high* degree of perfection can be easily attained. In this as in all other departments, he who best improves his opportunities will be the best man; but we mean that physical diagnosis shall be an open science, and not the property of a few expert specialists. Mr. Lee's essay is full of the idea which we would controvert, and, though it is the least of his sins, we cannot pass it without using it as a text to express our own belief on the practical value of physical exploration to the every-day practitioner.

We close that share of our article which we propose to devote to Mr. Lee, by referring to his statement that "auscultation and percussion form no part of the education of medical students" in this country. He makes this assertion as if he knows what he was saying—makes it with that positive, dogmatic John Bull-ism that admits of no contradiction. From whence, in the name of nonsense, did he derive his information? We are tempted to send him an announcement of the Medical Department of the University of Buffalo, wherein he may read that that branch of medical art is carefully and systematically taught at the bedside, that it is not taught orally, by lectures only, but by an accomplished Clinical Instructor, whose duty it is to attend to this laborious department, and who does so most successfully.

It may be that in some of the crowded institutions of our eastern cities it is impossible for the school to provide means of practical instruction, in its own faculty, for so many pupils; and that they are then, as in London, obliged to employ private instructors in order to secure sufficient training. This is undoubtedly the case, but it does not impair the fact that the means for clinical instruction are there, and the payment of a fee will secure them. This is a pecuniary, but not a positive disadvantage to the student; but it exists in London as much as in New York or Philadelphia. That it does not exist in Buffalo is an incident of a large faculty, abundant clinical means, and moderate classes.

But, taken in its intended meaning, the assertion of Mr. Lee is evidently a falsehood through gross ignorance, and with that censure we may dismiss it to turn our attention to the Rhode Island Medical Society.

When the committee awarded this prize, they knew that the author was English, but that of course, under their rules, should have made no difference. But they should not have awarded a prize to an essay containing gross mistatements: libellous of the profession, of America, and of American Medical schools. If they did not know the blundering ignorance of these allegations, they should have known it, and should not have given them the sanction of a State Society. We can imagine that a little vanity had something to do with this, and that the committee were flattered in an English competition for the prize. If so, this is only another instance of that lamentable toadyism which leads us eagerly to accept all Transatlantic theories for the unadulterated truth, and confide in them with a childish truthfulness. It is time we had done with this. We can never hope for a strong, vigorous American literature, so long as the American writer knows that, no matter how brilliant his discoveries, how accurate his analysis, or how philosophical his reasoning, he will be set down as necessarily inferior to foreign writers. The assumption is always that the European writer is the soundest. Druitt, a medical student, compiles his surgical notes, and, having the means, publishes them. His book is republished on this side, and bears the weight of a high authority; while in reality, at the time he wrote it, he had never amputated a limb or excised a tumor, and since then has failed to attain surgical distinction. Such instances are numerous, and we shall never see the last of them until American authors are met by the profession as, *prima facie*, as good as foreigners. And we are sorry and ashamed that a state society, even the diminutive state of Rhode Island, should have fallen into this national sin and blunder.

